# Prevalence of irritable bowel syndrome and its associated risk factors among university students of Bangladesh

**DOI:** 10.1002/jgh3.12757

**Published:** 2022-05-17

**Authors:** Anita Das, Arafat H Razon, Tanvir Ahmad, Dipak K Paul

**Affiliations:** ^1^ Department of Nutrition and Food Technology Jashore University of Science and Technology Jashore Bangladesh; ^2^ Department of Applied Nutrition and Food Technology Islamic University Kushtia Bangladesh

**Keywords:** body mass index, diet, irritable bowel syndrome (IBS), ROME III criteria

## Abstract

**Background:**

Irritable bowel syndrome (IBS) is a very common gastrointestinal disorder worldwide, but research regarding this disease is rare in Bangladesh. This study aimed to assess the prevalence of IBS and its associated risk factors among university students in Bangladesh.

**Methods:**

This is a cross‐sectional study. A total of 300 randomly selected participants were included in this study. By using a structured questionnaire and anthropometric methods, we collected all the required data for our study. The diagnosis of IBS was based on Rome III criteria.

**Results:**

The overall prevalence of IBS was 39.3%, but the majority (77.3%) had no basic awareness of IBS. In our study, anxiety and depression (*χ* ^2^ = 6.817; odds ratio [OR] = 1.910; 95% confidence interval [CI] = 1.172, 3.113; *P* = 0.011) had a significant relationship with IBS and IBS had a significant (*P* < 0.001) relationship with food intolerance (*χ* ^2^ = 8.737; OR = 2.130; 95% CI = 1.284, 3.531), chest pain (*χ* ^2^ = 7.482; OR = 2.035; 95% CI = 1.218, 3.401), and insomnia (*χ* ^2^ = 19.320; OR = 2.907; 95% CI = 1.794, 4.709). In our dietary data, the intake patterns of vegetables (*P* = 0.000), fast food (*P* = 0.000), and tea–coffee (*P* = 0.003) showed a strong significant association with IBS. On the other hand, monthly household income (*P* = 0.154) and body mass index (BMI) (*P* = 0.138) showed no significant association with IBS. Among our study subjects, IBS‐constipation (54.2%) was more common than IBS‐diarrhea (27.1%) and IBS‐mixed (18.6%). Moreover, among the 118 IBS respondents, 67.8% had a headache with increased flatulence (95.8%) as the most common IBS‐related complication.

**Conclusion:**

IBS is common in university students of Bangladesh and is associated with anxiety, depression, and particular dietary patterns.

## Graphical abstract

This is a cross‐sectional study of 300 randomly selected university student participants, determining the prevalence of “IBS” and various associated factors. The authors reported a 39.3% prevalence of “IBS” and that 77.3% were not aware of the condition. The authors then report various clinical, psychological, demographic, and dietary associations with “IBS” using univariate analysis.

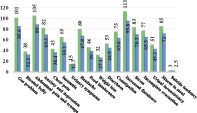

## Introduction

Irritable bowel syndrome (IBS) may be defined as one of the functional bowel disorders in which defecation and/or a change in bowel habit is linked with discomfort or abdominal pain. The most common characteristics of IBS are sensations of bloating, disordered defection, and distension. Around the world, “bloating” and “distension” are often recognized as the same term in various languages.^1^ Functional gastrointestinal disorders (FGIDs) can be identified by various symptom‐based diagnostic criteria, which involve mainly chronic or recurrent symptoms. Actually, until today, there is no known path‐physiological mechanism that accumulates these disorders. However, various studies have suggested some contributory factors that facilitate these disorders, and the most common factors are abnormal gastrointestinal motility, visceral hypersensitivity, altered brain‐gut function, low‐grade inflammation, psychosocial disturbance, and intestinal microbes.^2^

Additionally, dietary patterns of individuals are the most frequent influencer of IBS and food is the main topic of concern among IBS patients. Associations between food intake and increasing GI symptoms among IBS patients have been observed in various studies, and as a result of that, over the years, various dietary patterns have been used to minimize IBS symptoms among patients.^3^ Cultural dietary practices of various regions around the world are also responsible for the prevalence of IBS. For example, it is recommended to include at least 20–30 g of dietary fibers in our daily diet. However, it has been noted by previous studies that the Western diet contains a significantly lower amount of dietary fibers in their daily diet than the diet of the Indian subcontinent. As a result of that, the frequency of IBS is significantly higher among Western adults than in Indian adults.^4^ Although several studies have been conducted in India as well as Western countries regarding the effect of cultural dietary practices on the prevalence of IBS, dietary pattern‐related IBS studies are very limited in Bangladesh. For that reason, this study also focused on the relationship of the dietary pattern of the study subjects with the prevalence of IBS.

IBS has become a major GI problem among the population of the Asian region, especially it is more prevalent among countries like India and Bangladesh. Although these nations have many differences, they possess many similarities in their socio‐demographic, dietary, and cultural aspects.^5^ To identify the prevalence of IBS, various population‐based studies have been conducted among these nations, and the frequency of IBS varies from 4.2 to7.5% as well as from 7.7 to 12.9% between India and Bangladesh, respectively.^6^, ^7^, ^8^, ^9^, ^10^, ^11^ As Bangladesh has a comparatively higher prevalence rate of IBS than India, further studies among the Bangladeshi population are needed to justify their IBS condition. Moreover, several previous studies identified that IBS is highly predominant among university students worldwide. From a worldwide perspective, the prevalence of IBS among university students ranges from 5.7 to 34%, which includes 10.9% in the United States, 5.7% in Korea, 10.7% in Japan, 34% in Pakistan, 15.8% in Malaysia, and 26.1% in northern Nigeria.^12^, ^13^, ^14^ As there is a limited study on the prevalence of IBS among Bangladeshi university students, that is why through this study, we tried to illustrate the actual scenario regarding the prevalence of IBS and its associated risk factors among university students of Bangladesh.

## Methods

This descriptive cross‐sectional study was conducted from September until November 2020 among the university students aged 18–25 years at Jashore University of Science and Technology, Bangladesh.

The study protocol was reviewed and approved by the Ethical Review Committee, Faculty of Biological Science and Technology, Jashore University of Science and Technology, Bangladesh. The researchers conducted this study by following all the International Rules for Research Ethics. Information about the purposes and benefits of this research were properly explained to the participants and verbal consent was taken from all the participants. All the information given by the study participants was reserved under proper confidentiality. Students who had rapid weight loss with blood in stool were not included in this study. Female respondents who had gynecological issues such as postmenopausal syndrome (PMS) were excluded from this study to avoid the overlapping of IBS symptoms with PMS. The PMS state of the female respondents was confirmed by the university medical personnel. Any students who were unwilling to give information were excluded from this study, and the participants had the right to withdraw their participation anytime during this study. During our study, proper checking and supervision of the data for consistency and completeness were carried out.

A systematic random sampling method was used to collect data from the respondents. A predesigned structured interviewing questionnaire was developed to collect data from the respondents. The questionnaire was composed of various information such as personal information (name, sex, age), socio‐demographic information (religion, family member, monthly income), anthropometric information (height, weight, body mass index [BMI]), Rome III criteria, clinical information, awareness‐based information, and food frequency data of the respondents for the last 7 days. Before implementing the questionnaire in the study, we conducted a pilot survey to review, check, and revise the questionnaire properly.

The finally approved questionnaire was comprised of five sections. The first section of the questionnaire comprised of personal and demographic information related questions with options such as age, sex (male/female), religion (Muslim/Hindu), number of family members, family type (nuclear/joint), household monthly income in taka, awareness about IBS (Yes/No), and presence of IBS (Yes/No). Questions about the anthropometric information such as height, weight, and BMI were included in the second section of our questionnaire. We measured the weight of each respondent twice to the closest 0.1 kg by digital scale (Soehnle; CMS, London, UK) placed on a flat surface. In our analysis, the average of the two measurements was used. We measured the height of each respondent (without shoes) twice through the use of a Stadiometer (Cranlea Ltd., Birmingham, UK). In our analysis, we used the average of these measurements. BMI was calculated as weight (kg)/height (m^2^). Here in our study, we used Asian BMI cutoffs to define the nutritional status of our participants.^15^

In this study, IBS was diagnosed by a tool known as Roman criteria, and this criterion is mainly based on various symptoms to detect IBS. Three main Roman criteria have been developed for the diagnosis of IBS. The most refined criteria are Rome I and Rome II. Rome I and Rome II criteria are composed of the duration of IBS symptoms and some researchers have already argued that Rome II criteria have some limitations due to their restrictive nature. On the other hand, the Rome III criteria are the most flexible and reliable tool to diagnose IBS, but it is not a suitable IBS diagnosis tool for patients with red flag symptoms such as fever, vomiting, rectal bleeding, weight loss, etc. Besides these three Roman criteria, another additional Rome IV criteria are also used to diagnose IBS in which changes in bowel habit and abdominal pain are the major identical symptoms of IBS. In this study, IBS patients were mainly classified into four subgroups based on their stool patterns such as diarrhea‐predominant (IBS‐D)‐loose/watery stools happening at least 25% of defecations and lumpy or hard stools happening never or rarely, constipation‐predominant (IBS‐C)‐lumpy or hard stools happening at least 25% of defecations and watery/loose stools happening never or rarely, mixed diarrhea and constipation (IBS‐M)‐altering loose and hard stools, each happening at least 25% of defecations and patients who meet the diagnostic criteria for IBS, but whose bowel habits cannot be accurately categorized (IBS‐U)‐hard or loose stool happening never or rarely.^16^, ^17^, ^18^ In addition to that, the third section of the questionnaire was used for the diagnosis of IBS according to Rome III criteria and included some statements or opinions of the respondents in the context of Rome III criteria. Furthermore, here in this study, a locally validated Rome III questionnaire was used to diagnose IBS among the study subjects. The statements included the presence of frequent abdominal pain (Yes/No), abdominal discomfort (Yes/No), changes in the frequency and appearance of stool (Yes/No), improvement of defection (Yes/No) of the respondents for at least 3 days per month during the last 3 months.^18^

The fourth section of the questionnaire was for the assessment of awareness and clinical symptoms as well as associations of IBS among respondents. It included questions about the awareness of respondents regarding IBS as well as associations and clinical symptoms related questions such as the presence of weight loss, chest pain, insomnia, headache, anxiety, etc. The respondents were asked to respond to these questions in the form of Yes or No. In the fifth section, we used a food frequency questionnaire (FFQ) to determine the dietary habits of the participants. Each respondent was asked to remember what they ate during the last 7 days with a frequency option provided in the questionnaire to fulfill the FFQ, and this FFQ was used to determine the dietary intake pattern of the students.

We conducted all the statistical analysis through the use of the Statistical Package for Social Science (SPSS) windows version 21.0 (SPSS Inc., Chicago, IL, USA) software program. The sample size of this study was calculated through a simple equation, which is given below:

(1) $$N=Z^{2}\mathrm{pq}/d^{2}.$$

Here, *Z* = 1.96, *p* = 0.5 (as no study found), *q* = 0.5, and *d* = 0.05. The sample size was around 384 according to Equation (1); however, only 300 samples were collected for this study due to limitations of time and lack of funding. Data were entered into the software with proper care to avoid errors. Parametric and nonparametric analyses were performed for our variables as appropriate. Through the analysis, we expressed all the categorical variables as their percentage distribution. To identify the associations among our variables odds ratio (OR) with a 95% confidence interval (CI) test was performed and the significant associations of variables were expressed by the chi‐square test. In our study, *P* values smaller than 0.05 were considered statistically significant.

## Results

The basic characteristics of each respondent as well as their awareness of IBS are presented in Table 1. Variables of age, gender, marital status, family types, religion, family's monthly household income, respondents' awareness about IBS, and presence of IBS among respondents were expressed as frequency and percentage distribution. Almost 67% of our participants were female and the majority (84.7%) belonged to a nuclear family. Though approximately 61% of our respondents had the absence of IBS, a major portion, such as 77.3% of our respondents, had no basic awareness about IBS.

**Table 1:** Characteristics of the respondents (*n* = 300)

Variables	*n* (%)
Age (year)	
Male age (mean ± SD)	22.14 ± 2.089
Female age (mean ± SD)	21.45 ± 1.829
BMI	
Male BMI (mean ± SD)	22.005 ± 2.587
Female BMI (mean ± SD)	21.516 ± 2.663
Gender	
Male	100 (33.3%)
Female	200 (66.7%)
Marital status	
Unmarried	275 (91.7%)
Married	25 (8.3%)
Family type	
Nuclear	254 (84.7%)
Joint	46 (15.3%)
Religion	
Muslim	263 (87.7%)
Hindu	37 (12.3%)
Monthly household income	
<7000 taka	10 (3.3%)
7000–14 000 taka	44 (14.7%)
15 000–22 000 taka	118 (39.3%)
23 000–30 000 taka	64 (21.3%)
>30 000 taka	64 (21.3%)
Monthly expenses	
<2000 taka	12 (4.0%)
2000–3000 taka	186 (62.0%)
3100–4000 taka	63 (21.0%)
>4000 taka	39 (13.0%)
Awareness about IBS	
Yes	68 (22.7%)
No	232 (77.3%)
Presence of IBS	
Yes	118 (39.3%)
No	182 (60.7%)

BMI, body mass index; IBS, irritable bowel syndrome.

IBS had a significant relationship with associated symptoms like chest pain (OR = 2.035; 95% CI = 1.218, 3.401; *P* = 0.008), food intolerance (OR = 2.130; 95% CI = 1.284, 3.531; *P* = 0.004), and a significant association with anxiety and depression (OR = 1.910; 95% CI = 1.172, 3.113; *P* = 0.011). In case of weight loss, IBS had a significant (OR = 1.963; 95% CI = 1.113, 3.463; *P* < 0.005) relationship; moreover, on the other hand, urinal inconsistency also showed significant (OR = 2.387; 95% CI = 1.451, 3.927; *P* = 0.001) relationship with IBS (Table 2).

**Table 2:** Assessment of the association of non‐GI parameters with IBS among the respondents (*n* = 300)

	Irritable bowel syndrome		Total	*χ* ^2^	*P*	OR (95% CI)
Non‐GI parameters	Yes (%)	No (%)				
Anxiety and depression						
Yes	82 (69.5%)	99 (54.4%)	181 (60.3%)	6.817	0.011	1.910 (1.172–3.113)
No	36 (30.5%)	83 (45.6%)	119 (39.7%)			
Chest pain						
Yes	43 (36.4%)	40 (22.0%)	83 (27.7%)	7.482	0.008	2.035 (1.218–3.401)
No	75 (63.6%)	142 (78.0%)	217 (72.3%)			
Insomnia						
Yes	65 (55.1%)	54 (29.7%)	119 (39.7%)	19.320	0.000	2.907 (1.794–4.709)
No	53 (44.9%)	128 (70.3%)	181 (60.3%)			
Headache						
Yes	80 (67.8%)	109 (59.9%)	189 (63.0%)	1.920	0.180	1.410 (0.867–2.294)
No	38 (32.2%)	73 (40.1%)	111 (37.0%)			
Food intolerance						
Yes	46 (39.0%)	42 (23.1%)	88 (29.3%)	8.737	0.004	2.130 (1.284–3.531)
No	72 (61.0%)	140 (76.9%)	212 (70.7%)			
Weight loss						
Yes	32 (27.1%)	29 (15.9%)	61 (20.3%)	5.528	0.027	1.963 (1.113–3.463)
No	86 (72.9%)	153 (84.1%)	239 (79.7%)			
Stress						
Yes	83 (70.3%)	41 (22.5%)	124 (41.3%)	67.485	0.000	8.155 (4.818–13.804)
No	35 (29.7%)	141 (77.5%)	176 (58.7%)			
Urinal inconsistency						
Yes	51 (43.2%)	44 (24.2%)	95 (31.7%)	11.999	0.001	2.387 (1.451–3.927)
No	67 (56.8%)	138 (75.8%)	205 (68.3%)			

CI, confidence interval; GI, gastrointestinal; IBS, irritable bowel syndrome; OR, odds ratio; *χ* ^2^, Chi square.

**Figure 1:**
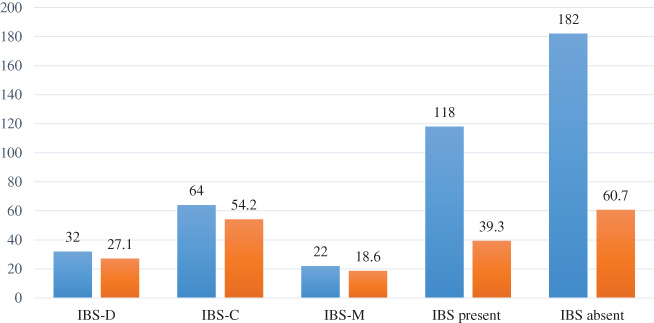
Characteristics and prevalence of irritable bowel syndrome among our study subjects (*n* = 300). 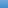, Frequency (*n*); 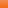, percentage.

Figure 1 illustrates the characteristics of IBS among our study population. Among the 300 respondents, we found that only 118 subjects had active IBS, and these respondents were significantly affected by three forms of IBS such as IBS‐D, IBS‐C, and IBS‐M. Although we found three forms of IBS among the respondents, the majority (54.2%) of them were affected by IBS‐C.

Among the 118 IBS respondents, we found that approximately 62% of them had normal BMI; however, we could not establish any significant (*χ* ^2^ = 5.511; *P* > 0.001) relationship between BMI with IBS. According to the dietary habits of our participants, we found a significant relationship between tea–coffee (*χ* ^2^ = 11.704; *P* = 0.003), vegetable intake (*χ* ^2^ = 37.559; *P* = 0.000), and soft drinks intake (*χ* ^2^ = 6.860; *P* = 0.032) pattern of our respondents with IBS (Table 3).

**Table 3:** Chi‐square test to estimate the association of various risk factors with the presence of irritable bowel syndrome among respondents (*n* = 300)

Irritable bowel syndrome				
Variables	Yes	No	*χ* ^2^	*P*
Monthly household income				
<7000	6 (5.1%)	4 (2.2%)		
7000–14 000	21 (17.8%)	23 (12.6%)		
15 000–22 000	46 (39.0%)	72 (39.6%)	6.683	0.154
23 000–30 000	27 (22.9%)	37 (20.3%)		
>30 000	18 (15.3%)	46 (25.3%)		
Body mass index (BMI)				
Underweight	15 (12.7%)	20 (11.0%)	5.511	0.138
Normal	73 (61.9%)	98 (53.8%)		
Overweight	16 (13.6%)	45 (24.7%)		
Pre‐obese	14 (11.9%)	19 (10.4%)		
Tea–coffee intake pattern				
Regularly	67 (56.8%)	72 (39.6%)	11.704	0.003
Irregularly	45 (38.1%)	83 (45.6%)		
Never	6 (5.1%)	27 (14.8%)		
Soft drinks intake pattern (per week)				
Regularly	28 (23.7%)	29 (15.9%)		
Irregularly	19 (16.1%)	51 (28.0%)	6.860	0.032
Never	71 (60.2%)	102 (56.0%)		
Vegetables intake pattern				
Regularly	45 (38.1%)	132 (72.5%)	37.559	0.000
Irregularly	64 (54.2%)	48 (26.4%)		
Never	9 (7.6%)	2 (1.1%)		
Fruits intake pattern				
Regularly	6 (5.1%)	34 (18.7%)		
Irregularly	61 (51.7%)	111 (61.0%)	23.792	0.000
Never	51 (43.2%)	37 (20.3%)		
Meat intake pattern				
Regularly	11 (9.3%)	42 (23.1%)		
Irregularly	100 (84.7%)	132 (72.5%)	9.386	0.009
Never	7 (5.9%)	8 (4.4%)		
Fast food intake pattern				
Regularly	33 (28.0%)	29 (15.9%)		
Irregularly	78 (66.1%)	74 (40.7%)	49.230	0.000
Never	7 (5.9%)	79 (43.4%)		
Salty snacks intake pattern				
Regularly	8 (6.8%)	7 (3.8%)		
Irregularly	74 (62.7%)	83 (45.6%)	11.974	0.003
Never	36 (30.5%)	92 (50.5%)		
Daily water consumption (100 mL/glass)				
<8	60 (50.8%)	66 (36.3%)		
8–10	50 (42.4%)	89 (48.9%)	8.265	0.016
>10	8 (6.8%)	27 (14.8%)		

*χ* ^2^, Chi square.

**Figure 2:**
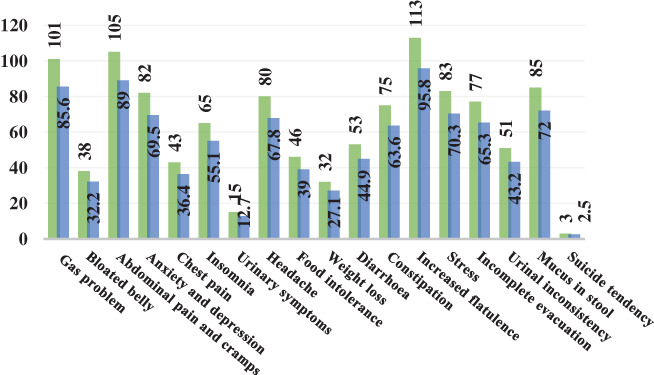
Overall scenario of irritable bowel syndrome‐related clinical symptoms and complications. 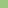, Frequency; 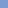, percent.

Figure 2 depicts the overall scenario of IBS along with its clinical symptoms and associations among our respondents. In our study, almost 40% of our participants had active IBS, and as a result of it, we noticed some major IBS‐related clinical symptoms and associations among them, such as anxiety and depression (69.5%), headache (67.8%), and stress (70.3%).

## Discussion

In our study, a total of 300 students from the Jashore University of Science and Technology were selected randomly to conduct this study. By investigating our research data, it was acknowledged that 39.3% of respondents were suffering from IBS according to Rome III criteria, and this result is not so consistent with a study conducted in New Delhi, India among medical students, where they found only 16.5% prevalence of IBS among them.^19^ In comparison with previous studies, a very high prevalence of IBS has been noticed among this study subjects, which may be due to the use of Rome III criteria to diagnose IBS because the majority of previous IBS‐related studies have used Rome II criteria, which have less accuracy to diagnose IBS patients than Rome III criteria. According to some recent studies, the prevalence of IBS has become one of the most common disorders in females than in males, with a female‐to‐male ratio of 2–2.5:1. However, in the Asian region, this scenario is different where the ratio of IBS patients among males and females is almost equal.^20^ Although the results of our study showed some variation from other Asian reports, we noted that female respondents were more likely to suffer from IBS than male respondents; a finding in line with many reports of IBS throughout the world. Besides the prevalence of IBS, we surprisingly noticed that majority of our participants did not have proper awareness about IBS, and it was observed from the analysis of our survey data that approximately 78% of our respondents had poor awareness regarding IBS. Our study result is contrasted with the study, which was conducted in Riyadh city of Saudi Arabia from October to December 2017, and in their study, they mentioned that only 18.9% of their study population had poor awareness regarding IBS.^21^

From our study, we noticed a significant relationship between IBS and depression, as well as we noticed a significant relationship between daily water intake and IBS. This finding is not so consistent with another study where an insignificant association was found between whole‐day water consumption and odds of IBS among the adult population.^22^ From the analysis of our survey data, we found that 118 respondents had active IBS, and among them, the most predominant (54.2%) category of IBS was IBS‐C. Our study result depicts a variation with a recently published US population‐based survey where 37.3% of patients were diagnosed with IBS‐C.^23^

The monthly household family income of our respondents did not show any significant association with their IBS condition. So, for our study population, household family income may not be a cause of IBS condition, and this report is matched with a previous study where IBS was not a big problem in lower socioeconomic groups.^24^

We tried to interpret a relationship between BMI and IBS, but we did not find any significant association between them. However, by reviewing previous IBS‐related studies, we noticed that majority of them mentioned a positive association between obesity and IBS.^25^, ^26^

The current study concluded a significant association between the presence of IBS and the vegetable intake pattern of the respondents, which is an expression of the ability of vegetables to lower the symptoms of IBS. This finding is supported by another study, which stated an IBS lowering activity of vegetables as well as mentioned a significant association of IBS with fatty food consumption.^27^ Our study showed that soft drinks consumption had an association with IBS, and our study participants reported that after consuming soft drinks, they faced various bowel and gas problems. In the present work, we noticed that tea–coffee consumption had a significant association with IBS, and IBS was more prevalent among those who consumed tea–coffee regularly. A similar study about the prevalence of IBS in Turkey observed that IBS was higher among individuals who did not take their meals regularly and did not consume a sufficient amount of fiber in their diet. Moreover, the study also revealed that daily consumption of cola increased the prevalence of IBS among Turkish respondents; however, on the other hand, it could not establish any significant association between IBS and daily intake of tea or coffee.^28^ From the overall scenario, it was observed from this study that our study population not only suffered from diarrhea and constipation‐predominant IBS but also highly suffered from flatulence predominant IBS, which is comparable with a study conducted in India among medical students.^19^

## Conclusion

Based on the present study results, IBS‐C was the most prevalent type of IBS among our respondents, and besides this, majority of our respondents did not have basic awareness about IBS. In terms of the dietary pattern of our subjects, vegetable consumption was found to lower the risk of IBS; however, the severity of IBS was increased highly among respondents who ate fast foods regularly. From this study, we have observed that IBS not only caused physiological problems but also psychological problems, which negatively affected the quality of life. To minimize these IBS‐related physiological and psychological problems, proper knowledge about this disease is mandatory. Proper nutritional counseling will be needed to be introduced.
